# Inhibition of BIRC5 and MCL-1 as a potential treatment strategy to overcome drug resistance in Mantle Cell Lymphoma

**DOI:** 10.1038/s41408-026-01467-1

**Published:** 2026-03-11

**Authors:** Jeremiah Pfitzer, Sayak Chakravarti, Suman Mazumder, Fengzhi Li, Ujjal Kumar Mukherjee, Amit Kumar Mitra

**Affiliations:** 1 Department of Drug Discovery and Development, Harrison College of Pharmacy, Auburn University, Auburn, AL USA; 2 Center for Pharmacogenomics and Single-Cell Omics Initiative, Auburn University, Auburn, AL USA; 3 Department of Pharmacology & Therapeutics, Roswell Park Comprehensive Cancer Center, Elm and Carlton Streets, Buffalo, NY USA; 4 Department of Business Administration, University of Illinois at Urbana-Champaign, Champaign, IL USA; 5 Carle Illinois College of Medicine, University of Illinois at Urbana-Champaign, Champaign, IL USA

**Keywords:** B-cell lymphoma, Drug development

Dear Editor,

Mantle cell lymphoma (MCL) is a highly heterogeneous and aggressive form of non-Hodgkin lymphoma that develops from malignant B-lymphocytes in the outer edge (mantle zone) of lymph nodes [1]. Despite promising initial responses to conventional chemotherapeutic drugs and targeted therapies like Bruton’s tyrosine kinase inhibitor (BTKi) and proteasome inhibitors (PI), patients develop resistance over time, thus progressing into more aggressive, incurable, refractory or relapsed (R/R) disease states, with a median progression-free survival of ~15 months [1]. We have designed a novel pharmacogenomics data-driven computational pipeline that utilizes a modified greedy algorithm (minimal set-cover optimization followed by regularization) to predict molecular targets for the development of small-molecule inhibitors as secondary drug candidates (‘secDrugs’) for the management of treatment-resistant cancers [2]. Previously, we demonstrated that the top-predicted secDrugs show significant cytotoxicity against in vitro and ex vivo models of advanced-stage cancers, including relapsed/resistant multiple myeloma and lethal variants of prostate cancer [2, 3]. Since the top among the predicted secDrug targets in drug-resistant B-cell malignancies included BIRC5 and MCL-1, we hypothesize that drug combinations utilizing BIRC5-inhibition and/or MCL-1-inhibition will be useful in curbing oncogenic progressions of MCL and abrogating drug resistance through simultaneous inhibition of multiple pathways [2]. Therefore, we selected YM155 (BIRC5 inhibitor) and S63845 (MCL-1 inhibitor) for our proof-of-concept experiments in MCL.

## Results

**Fig. 1:**
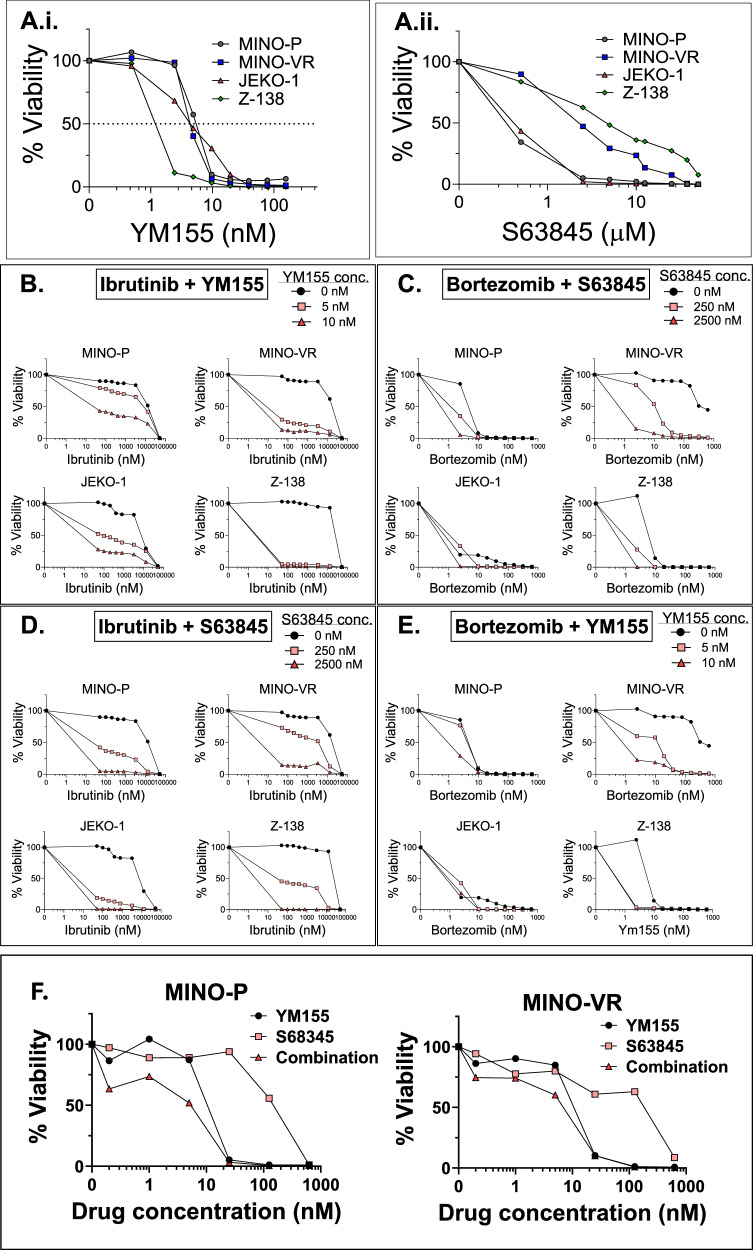
In vitro cell viability assays. **A** Dose-response plots in MCL cell lines showing single-agent in vitro cytotoxicity of (i) YM155 and (ii) S63845. YM155 & S63845 exhibit synergistic cell-killing activity when combined with PI and BTKi. Dose-response plots representing the in vitro cell viability profile of MCL cell lines, including PI-resistant and BTKi-resistant MCL cell lines, treated with different combinations of **B**, **C** YM155 and **D**, **E** S63845 with PI and BTKi. **F** Combination of secDrugs (BIRC5-inhibitor + MCL-1-inhibitor) has a synergistic effect. Representative plots showing secDrug-secDrug (YM155 + S63845) combination therapy analysis in sensitive and resistant MCL cell lines. The graphs represent percentage cell viability compared to no-treatment control. The combination index (CI) and Dose reduction index (DRI) values were calculated by Calcusyn Software (BioSoft) using Chou-Talalay’s algorithm (CI < 0.9 indicates drug synergy).

**Fig. 2:**
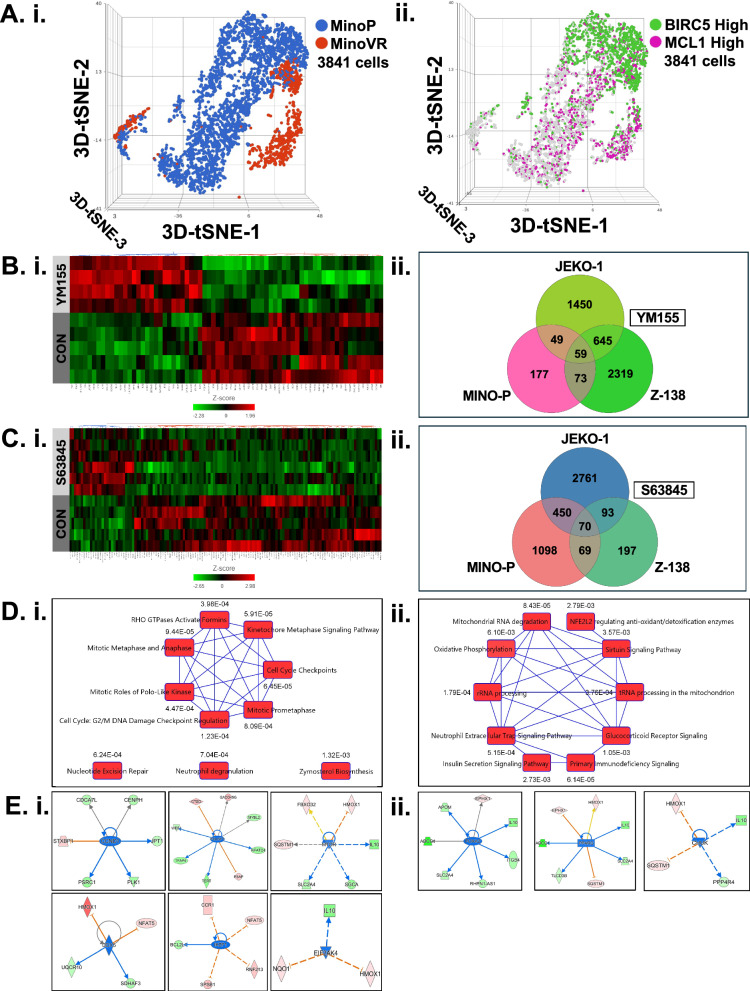
Single-cell and bulk tumor transcriptome analysis. **A** Baseline single-cell RNAseq analysis on the MCL cell line pair MINO-P/VR (*n* = 3841 cells). (i) Comparison of the t-SNE/Graph-based clusters between MINO-P vs MINO-VR cell lines. (ii) Figures showing single cells with enriched expression of the target genes BIRC5 and MCL-1; **B**, **C** Pre-vs-Post treatment RNAseq: (i) Heatmaps depicting the genes that were most significantly differentially expressed following treatment in the 4 MCL cell lines representing PI/BTKi sensitivity (JEKO1, MINO-P), innate resistance (Z-138), and clonally-derived acquired resistance (MINO-VR). (ii) Venn Diagrams showing the unique and shared DEG signatures between the MCL cell lines. **B** YM155 and C. S63845; **D**, **E** Ingenuity Pathway Analysis based on the list of significantly differentially expressed genes (DEGs) predicted secDrug-induced **D**. Canonical pathways and **E**. Upstream regulators. (i) YM155 (ii) S63845.

Table S1 lists the reagents used in our study. First, we evaluated the in vitro cytotoxicity of the secDrugs, YM155 and S63845, as single agents against a panel of MCL cell lines (Fig. S1) representing PI/BTKi sensitivity (JEKO1, MINO-P), innate resistance (Z-138), and clonally-derived acquired resistance (MINO-VR). We demonstrated that these secDrugs effectively reduced cell viability in all MCL lines, irrespective of PI/BTKi sensitivity/resistance (Fig. 1A). The half-maximal inhibitory concentration (IC_50_) ranges of single-agent YM155 and S63845 were 1.57–5.64 nM and 250 nM–4.7 µM, respectively. Further, combination treatment regimens of YM155 and S63845 with increasing dose ranges of PI (Bortezomib (BTZ/Bz/Velcade/V)) or BTKi (Ibrutinib) showed higher cytotoxic effects than single-agent PI/BTKi treatment (Fig. 1B–E). Combination index (CI) values calculated using Chou-Talalay’s theorem were consistently <0.7 (CI < 0.9 indicates synergism). Synergistic effects were particularly profound in cell lines representing R/R MCL (MINO-VR and Z-138). Notably, the median Dose Reduction Index (DRI) for Ibrutinib in combination with YM155 and S63845 was 26.28 and 62.60, respectively, and for Bortezomib was 7.95 and 52.52, respectively, which indicates that adding YM155 or S63845 significantly lowered the effective dose of BTKi and PI. Supplementary Table S2 summarizes the CI and DRI values corresponding to the combination experiments shown in Fig. 1B–E. Most importantly, we observed that the combination of these two secDrugs (YM155 + S63845) has substantial synergistic cell-killing action in MCL cells (Fig. 1F) (CI_IC50_ = 0.550; DRI_YM155_ = 2.454; DRI_S63845_ = 7.032; Fig. S2). Loss of cell viability due to apoptosis in response to secDrug single-agent and combination treatments was confirmed using Fluorescein isothiocyanate (FITC)-conjugated Annexin-V staining, followed by flow cytometry, and Caspase 3/7 activity assay (Fig. S3A–B). JC-1 is a cationic carbocyanine dye that accumulates in the matrix of healthy mitochondria and forms J-aggregates, which emit red fluorescence. Its accumulation decreases in depolarized mitochondria of apoptotic cells, and JC-1 remains in a monomeric form, emitting green fluorescence. Thus, a decrease in the ratio of red/green fluorescence indicates mitochondrial depolarization. We observed a significant shift from red to green fluorescence in response to YM155 and S63845 treatment, indicating mitochondrial dysfunction/depolarization due to loss of mitochondrial membrane potential (ΔΨ), which confirms that these secDrugs augment apoptosis in MCL cells via mitochondrial-mediated pathway (Fig. S3C). Aldehyde dehydrogenase (ALDH) is an intracellular detoxification enzyme frequently overexpressed in cancer stem cells (CSCs) and involved in drug resistance and aggressiveness in several cancers [4]. We observed >10-fold higher ALDH activity in the drug-resistant MCL lines compared to the drug-sensitive MCL lines. Furthermore, as BIRC5 expression overlaps with stem cell markers, YM155 single-agent treatment resulted in >90% reduction in ALDH activity in resistant MCL cells compared to the untreated control (Fig. S4). To integrate -omics-based analysis with our drug prediction pipeline, we used single-cell RNA sequencing (scRNAseq) as a novel biomarker-based drug screen and showed that majority of the single-cell subclones (*n* = 3841 cells; 89,856,511 paired-end reads) have high baseline expression of BIRC5 and MCL-1 (Fig. 2A), confirming that inhibition of these targets may be an effective treatment strategy against these subpopulations. The heatmaps and Venn Diagrams in Fig. 2B, C represent the results of bulk tumor RNAseq analysis of secDrug treatment-induced (pre-vs-post-treatment) changes in MCL cell lines. 1221 genes were significantly differentially expressed (DEGs) (p < 0.05;fold-difference≠1) following YM155 single-agent treatment. Among these, 857 genes had a |fold-change | ≥1.5 (Fig. 2B.i). When cell line-wise analysis was performed, 59 DEGs were shared at |fold-change | ≥1.5 (Fig. 2B.ii). As shown in Fig. 2C.i, the DEG signature of single-agent S63845 consists of 367 genes (p < 0.05;fold-difference≠1). Among these, 132 genes had a |fold-change | ≥ 1.5. 70 were shared between the MCL cell lines (Fig. 2C.ii). The top 50 DEGs for each single-agent treatment (YM155/S63485) are listed in Table S3.A–B. The following 5 DEGs were common between YM155 and S63845 single-agent treatments: ABCG1, EPHX1, SQSTM1, GADD45G, and NQO1. NQO1 and GADD45G are genes with tumor-suppressing and apoptosis-inducing functionality in response to cellular stress [5]. Interestingly, several top genes included in the single-agent DEG signatures exhibited further dysregulation following combination treatment, implying possible mechanisms of drug synergy. Table S4.A–D lists the top DEGs for YM155 + BTZ, YM155 + IBR, S63845 + BTZ, and S63845 + IBR. Based on the top DEGs, Ingenuity Pathway Analysis (IPA) predicted that YM155 induces up-regulation of pro-apoptotic markers, down-regulation of pro-survival genes, and several pathways associated with cancer aggressiveness and oncogenic progression, including mTOR signaling and genes linked to mitosis, cell cycle checkpoints, and chromosomal stability, like the mitotic regulators AURKB (Aurora kinase), NDC80, SPC24, CENPV, PIMREG, and CENPH (Fig. 2D.i). Mitochondrial dysfunction and oxidative phosphorylation were the top pathways for S63845 (Fig. 2D.ii). S63845 treatment resulted in significant upregulation of several mitochondrial genes, MT-CYB, MTND-2, and MTNDP424, suggesting a compensatory response to mitochondrial stress induced by MCL-1 inhibition, as MCL-1 is known to affect mitochondrial integrity [6]. S63845-induced increase in SQSTM1 and FBXO32 is indicative of enhanced autophagy and proteasomal degradation, suggesting tumor suppressor effects [7, 8]. We also observed increases in ferroptosis-related genes (HMOX1, SNORD3A), which are likely up-regulated as cellular defense against DNA damage and oxidative stress [9]. Further, IPA predicted downregulation of the following upstream regulators following secDrug treatment: DDX5, CCND1, Ikaros (IZKF1), EIF2AK4, PPARG, and FOXA1 (Fig. 2E). The on-target effect of secDrugs in MCL cells was validated using immunoblotting assays. Interestingly, although we observed that YM155 downregulates the expression of BIRC5 protein, treatment of MCL cells with S63845 increased the expression of its target protein, MCL-1, but not MCL-1 mRNA (Fig. S5). This has also been observed earlier, where S63845 treatment-induced increase in MCL-1 protein level was shown to be correlated with protein half-life [10]. Finally, we used gene expression data (GSE141335) on primary samples from patients with MCL and compared the association of our treatment-induced DEGs with the gene signatures of ex vivo IBR sensitivity. Our results show that several genes that were dysregulated by YM155 and S63845 treatment were also significantly associated with drug response, tumor survival, apoptosis (DNPH1), cell proliferation, differentiation, oxidative phosphorylation, mitochondria-regulated pathways, cancer progression, drug resistance, metastasis (ATP7B, ATP5F1C; COMTD1), and autophagy (WHAMM) in patients, indicating that our secDrugs are capable of reversing the oncogenic progression and drug resistance in MCL (Fig. S6A). Additional in silico validation of the differentially expressed genes associated with S63845 and YM155 treatment was conducted using The Cancer Genome Atlas’s (TCGA) Diffuse Large B-cell lymphoma (DLBC) gene expression profiling dataset. Our results show that several top DE genes for YM155 and S63845 were significantly associated with clinical outcomes, i.e., overall survival and disease-free survival (Fig. S6B).

## Discussion

BIRC5 is a member of the inhibitor of apoptosis (IAP) protein family that inhibits apoptosis through caspase-dependent and independent pathways, blocks cell death, and influences resistance to anti-cancer therapies [11]. MCL-1 is a pro-survival protein that belongs to the BCL-2 family of proteins, which are characterized by the presence of BCL-2 homology (BH) domains [10]. MCL-1 regulates the mitochondrial apoptotic pathway and has been implicated in the growth of tumors in several cancers, while the downregulation of MCL-1 may lead to cytotoxicity in tumor cells [12]. Interestingly, BIRC5 and MCL-1 have both been reported to be over-expressed and strongly correlated with progression and survivability of patients with B-cell lymphomas, and their inhibition has shown potency in several hematological malignancies [13–15]. Here, we confirmed our secDrug predictions by showing that inhibition of BIRC5/Survivin (YM155) and MCL-1 (S63845 – a BH3 mimetic) exhibited significant cell-killing activities as single agents and in combination with Bortezomib/PI and Ibrutinib/BTKi, especially in R/R MCL cells. Notably, we show the benefit of combining these two secDrugs (YM155 + S63845) as an effective dual-inhibition strategy against MCL. We also observed that YM155 is remarkably effective in reducing ALDH activity in resistant MCL cells. ALDH + MCL cells are found to be highly clonogenic, relatively quiescent (‘cancer stemness’), and resistant to chemotherapy [4]. Next, using single-cell and bulk tumor RNAseq, and cell-based assays, we identified genes and molecular networks associated with the mechanism of secDrug action and drug synergy. Finally, in silico analysis using patient datasets underlined the clinical potential of these candidates in curbing oncogenic progression and drug resistance. In summary, our study highlights the importance of BIRC5 and MCL-1 as novel therapeutic targets in aggressive forms of MCL, and provides additional novel drug candidates to overcome resistance to standard-of-care treatments.

## Supplementary information

Supplementary Figures and Tables

## Data Availability

All RNAseq data generated during the current study are available at GEO under accession number GSE312789. Public data were obtained from GSE141335.
