# Supplementary material for: Inhibition of BIRC5 and MCL-1 as a potential treatment strategy to overcome drug resistance in Mantle Cell Lymphoma
Source: Blood Cancer J. 2026 Mar 11;16(1):28. doi: 10.1038/s41408-026-01467-1 (PMC12979630; doi:10.1038/s41408-026-01467-1)
Supplement: Supplementary file 1 — Supplementary Figures and Tables [file 41408_2026_1467_MOESM1_ESM.docx]

Supplementary Materials for

**Inhibition of BIRC5 and MCL-1 as a potential treatment strategy to overcome drug resistance in Mantle Cell Lymphoma**

Pfitzer, Chakravarti *et al*

*Corresponding author. Email: akm0060@auburn.edu

**This file includes:**

Figs. S1 to S6

Tables S1 to S4

Fig. S1. Single-agent cytotoxicity assay with proteasome inhibitor (PI) and Bruton's Tyrosine kinase inhibitor (BTKi) shows extensive inter-individual variation in drug response in our MCL cell line panel. Dose-response curves reveal a wide range of drug sensitivity towards the PI Bortezomib (BTZ; Figure S1A) and the BTKi Ibrutinib (IBR; Figure S1B). MINO-VR has an approximately 70-fold higher IC_50_ value for Bortezomib than its parental cell line, MINO-P. Z-138 has an approximately 2.5-fold higher IC_50_ value for Ibrutinib than MINO-P and JEKO-1.

**S1B.**

**S1A.**


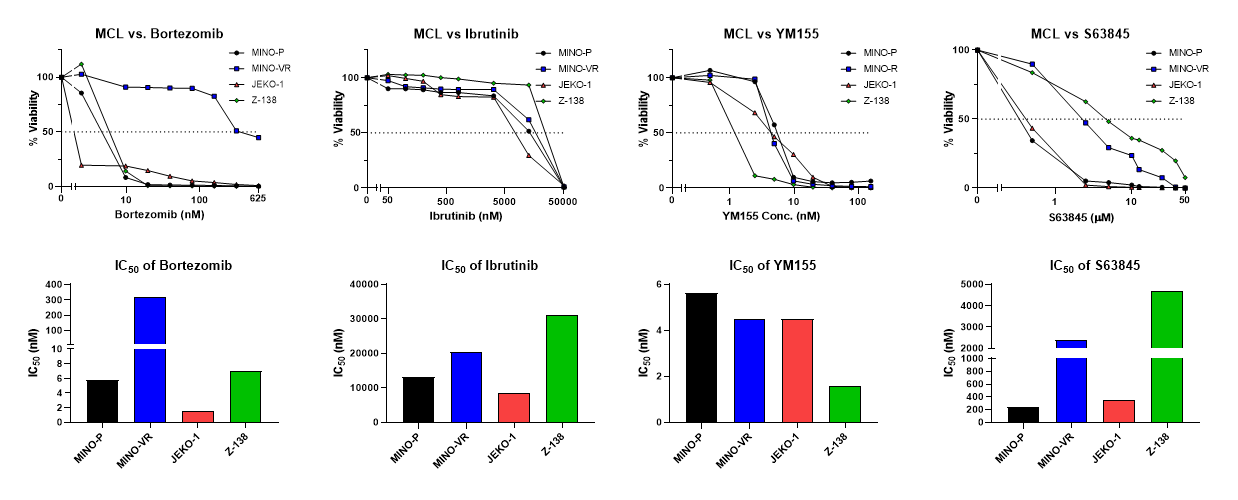


Fig. S2. Representative isobologram depicting secDrug-secDrug (YM155 + S63845) combination therapy in MCL cell line. Results show that the YM155+S63845 combination has synergistic activity (CI<0.9; DRI>1). The combination index (CI) and Dose reduction index (DRI) values were calculated by Calcusyn Software (BioSoft) using Chou-Talalay's algorithm (CI<0.9 indicates drug synergy).


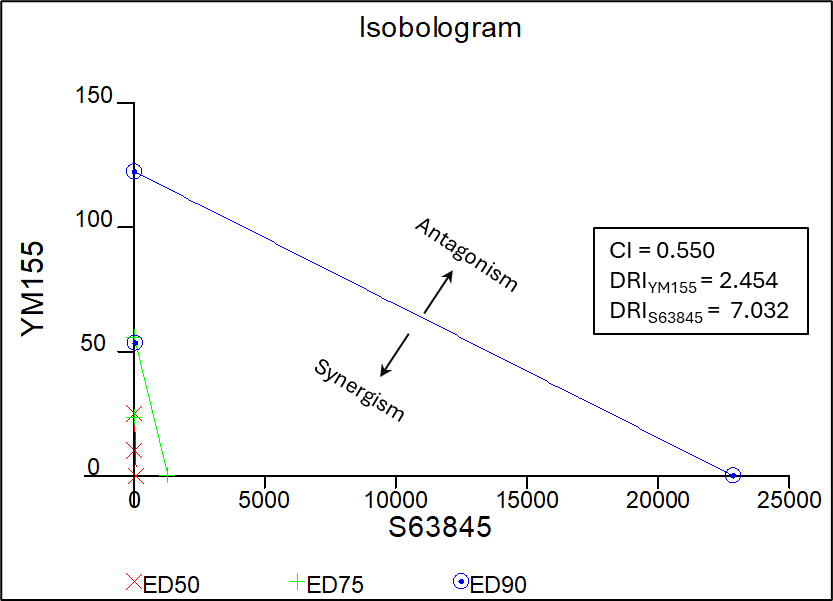


**Figure S3. secDrugs augment apoptosis in MCL cells via mitochondrial-mediated pathway. A)** Apoptotic cells representing the effect of secDrug on MCL cell lines as single agent and in combination with BTKi. Cells were treated for 48 hours, and then apoptosis was measured using Annexin V-FITC and flow cytometry. Results show a significantly higher population of cells that are Annexin V positive in combination treatment as compared to single agent treatment, indicating an elevated level of induction of apoptosis. **B)** Representative Caspase 3/7 activity assays showing increased apoptosis following YM155 and S63845 single-agent and combination treatment. **C)** secDrug treatment affects mitochondrial membrane potential (MMP) in MCL cells. Representative plots confirm the decrease in mitochondrial membrane potential following YM155 and S63845 treatment. JC-1 (Abcam), a cationic carbocyanine dye that accumulates in healthy mitochondria and forms aggregates, was used to assess Mitochondrial membrane potential (∆Ψ_M_ ), and fluorescence was recorded in a Synergy Neo 2 Microplate Reader (BioTek; Winooski, VT, US). (* P<0.05; ** P<0.01; *** P<0.001; **** P<0.0001)


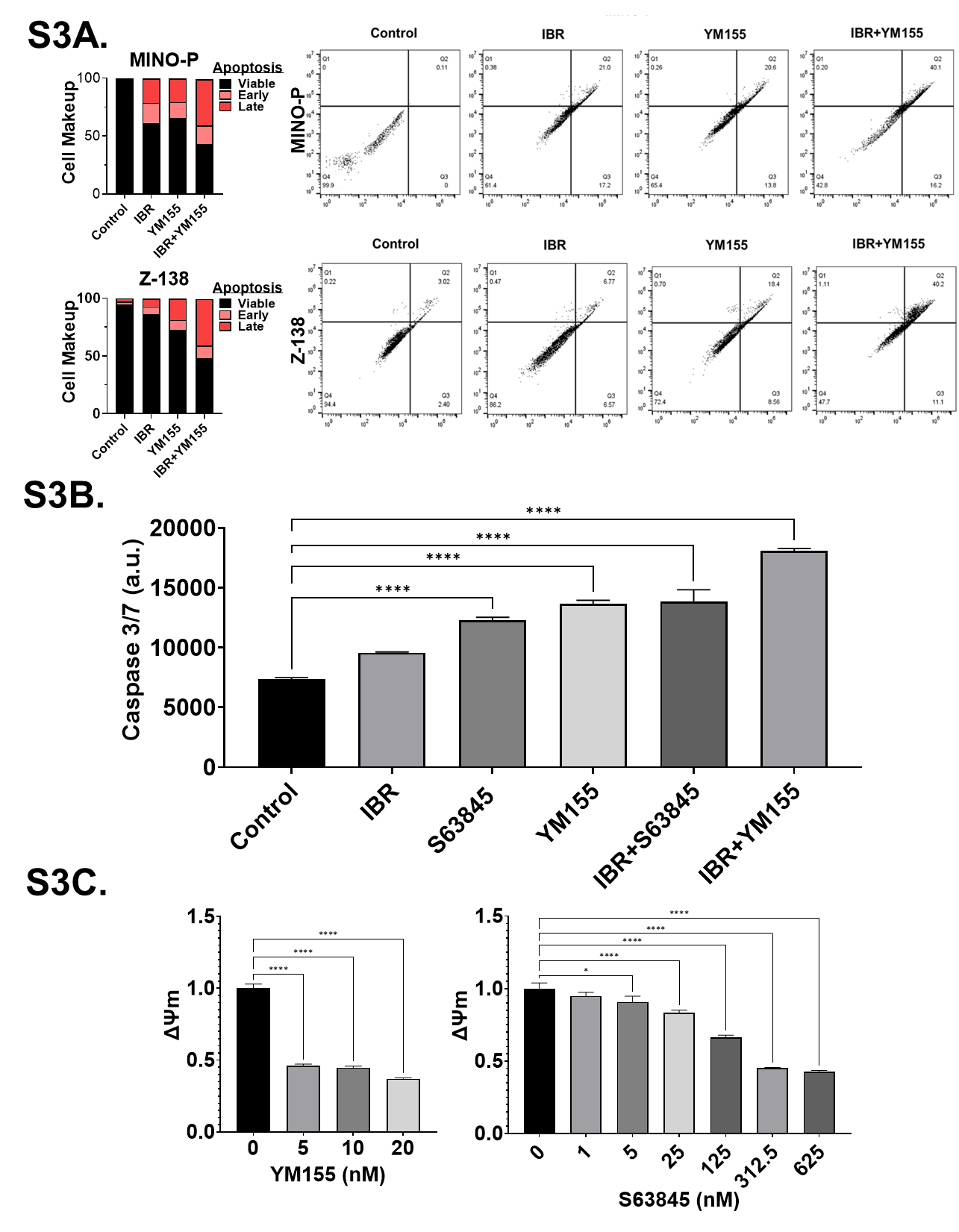


**Figure S4. Aldehyde dehydrogenase (ALDH) activity in MCL cell lines following YM155 treatment.** YM155 treatment led to a significant decrease in ALDH activity (>90%) in drug-resistant Z-138 cells as compared to single-agent Ibrutinib treatment (∼29%). N,N-diethylaminobenzaldehyde (DEAB) - a reversible, competitive inhibitor of ALDH, was used as a positive control.


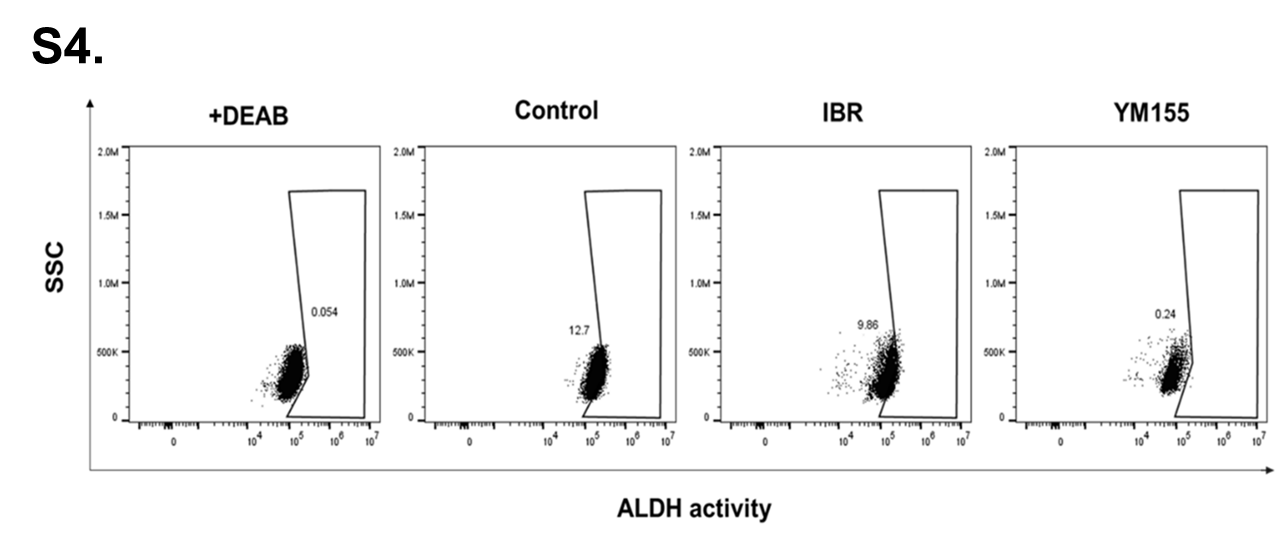


**Figure S5. Target validation.** Representative western blots show **A)** YM155 significantly down-regulates Survivin/BIRC5 protein, and **B)** BIRC5 mRNA expression; **C**) S63845 increases MCL-1 protein expression, and **D)** S63845 has minimal effect on MCL-1, BAX, and BCL-xL mRNA levels in MCL cells. (* P<0.05; ** P<0.01; *** P<0.001; **** P<0.0001)


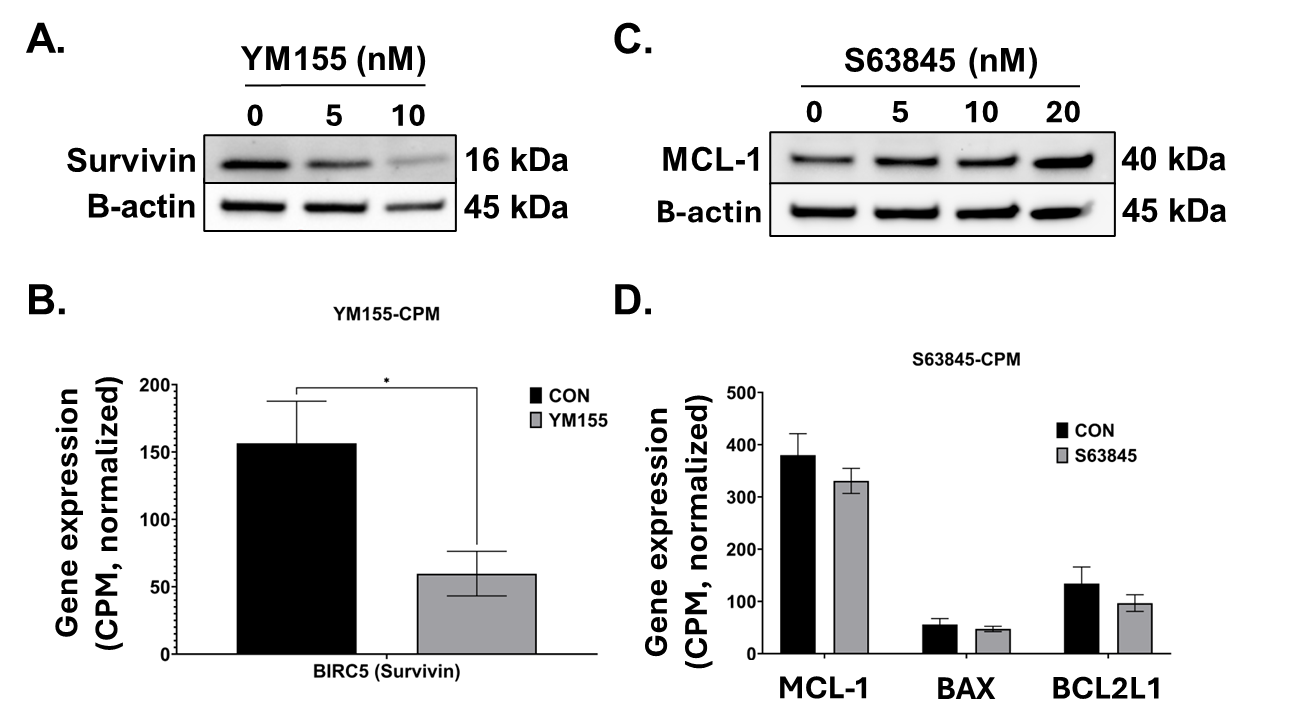


**Figure S6. *In silico* validation using secDrug treatment signatures. A)** *Ex vivo* IBR sensitivity vs gene expression plots for secDrug DEGs in primary samples from MCL patients (GSE141335). Patients in the cohort who were treated with the BRD4 inhibitor or NVP2 were excluded from our analysis. **B)** Kaplan-Meier plots showing secDrug treatment-associated genes are significantly associated with survival in lymphoma patients from the TCGA database. i) YM155 gene signatures. ii) S63845 gene signatures.


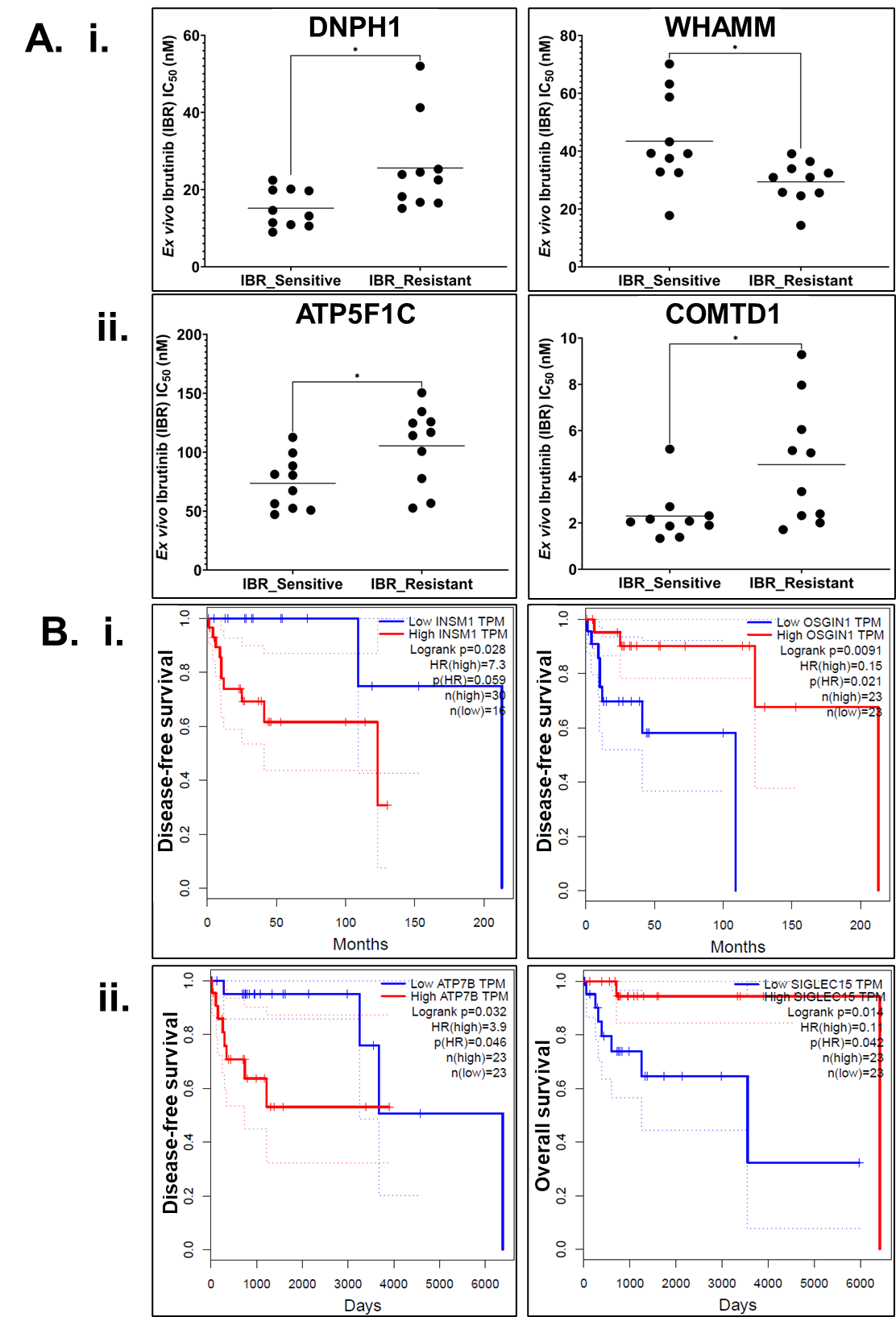


**Table-S1.** **List of drugs, reagents, antibodies, and kits used in the study.**

| **Reagents** | **Manufacturer** | **Location** |
| --- | --- | --- |
| **RPMI-1640 Medium, American Type Culture Collection** | ATCC (American Type Culture Collection) | Manassas, VA, USA |
| **Fetal bovine serum (FBS)** | Hyclone (Thermo-Fisher Scientific Inc.) | Rockford, IL, USA |
| **Recombinant Human IL-6** | PeproTech, Inc. | Cranbury, NJ, US |
| **Penicillin-Streptomycin (10,000 U/mL)** | Gibco^TM^ (Thermo-Fisher Scientific Inc.) | Waltham, MA, USA |
| **FITC Annexin V Apoptosis Detection Kit** | BD Biosciences | San Jose, CA, USA |
| **RIPA lysis buffer** | Thermo-Fisher Scientific Inc | Waltham, MA, USA |
| **Halt Protease and Phosphatase Inhibitor Cocktail** | Thermo-Fisher Scientific Inc | Waltham, MA, USA |
| **Pierce ECL Western Blotting Substrate** | Thermo-Fisher Scientific Inc | Waltham, MA, USA |
| **RNeasy Plus Mini Kit** | QIAGEN | Hilden, Germany |
| **Quick Start Bovine Serum Albumin Standard** | Bio-Rad | Hercules, CA, USA |
| **Tris Buffer Saline (TBS)** | Bio-Rad | Hercules, CA, USA |
| **10% Tween 20** | Bio-Rad | Hercules, CA, USA |
| **Polyvinylidene fluoride membrane (PVDF)** | EMD Millipore | Billerica, MA, USA |
| **Bovine Serum Albumin (BSA)** | VWR | Radnor, PA, USA |
| **Dimethyl sulfoxide (DMSO)** | Sigma-Aldrich Inc | St. Louis, MO, USA |
| **Bradford Reagent** | Sigma-Aldrich Inc | St. Louis, MO, USA |
| **JC-1 - Mitochondrial Membrane Potential Assay Kit** | Abcam | Waltham, MA, USA |
| **Anti-rabbit IgG, HRP-linked Antibody (7074S)** | Cell Signaling Technology | Danvers, MA, USA |
| **β-actin (A3854)** | Sigma-Aldrich Inc | St. Louis, MO, USA |
| **Calcein AM, cell-permeant green and blue dyes** | Thermo-Fisher Scientific Inc | Waltham, MA, USA |
| **S63845** | Selleckchem | Houston, TX, USA |
| **YM155** | Selleckchem | Houston, TX, USA |
| **MCL-1 antibody** | Cell Signaling Technology | Danvers, MA, USA |
| **Survivin antibody** | Cell Signaling Technology | Danvers, MA, USA |
| **Cyclin D1 (CCND1) antibody** | Cell Signaling Technology | Danvers, MA, USA |
| **β-actin antibody** | Cell Signaling Technology | Danvers, MA, USA |
| **Cleaved caspase-3** | Santa Cruz Biotechnology | [Dallas, TX](https://www.google.com/search?rlz=1C1CHBF_enUS878US878&sxsrf=AOaemvJo-09jZQ_VsP8vLx4tPHi-ch8TEQ:1636240031131&q=Dallas&stick=H4sIAAAAAAAAAOPgE-LUz9U3SDPOTUpT4gAzjYoKtbSyk63084vSE_MyqxJLMvPzUDhWGamJKYWliUUlqUXFi1jZXBJzchKLd7AyAgA6bbTQTwAAAA&sa=X&ved=2ahUKEwjo_smO7YT0AhWlSt8KHTMyCQ8QmxMoAXoECEAQAw), USA |
| **Cleaved caspase-9** | Santa Cruz Biotechnology | [Dallas, TX](https://www.google.com/search?rlz=1C1CHBF_enUS878US878&sxsrf=AOaemvJo-09jZQ_VsP8vLx4tPHi-ch8TEQ:1636240031131&q=Dallas&stick=H4sIAAAAAAAAAOPgE-LUz9U3SDPOTUpT4gAzjYoKtbSyk63084vSE_MyqxJLMvPzUDhWGamJKYWliUUlqUXFi1jZXBJzchKLd7AyAgA6bbTQTwAAAA&sa=X&ved=2ahUKEwjo_smO7YT0AhWlSt8KHTMyCQ8QmxMoAXoECEAQAw), USA |
| **Monoclonal Anti-β-Actin−Peroxidase (Mouse)** | Sigma-Aldrich | St Louis, MO, USA |
| **Anti-mouse IgG, HRP-linked Antibody** | Cell Signaling Technology | [Danvers, MA](https://www.google.com/search?rlz=1C1CHBF_enUS878US878&sxsrf=AOaemvIga4EeEji4x9E7mXFgVRfe0zLDKQ:1636240098401&q=Danvers,+Massachusetts&stick=H4sIAAAAAAAAAOPgE-LSz9U3MCu3KKlIUeIEsQ1zKsrztLSyk63084vSE_MyqxJLMvPzUDhWGamJKYWliUUlqUXFi1jFXBLzyoAsHQXfxOLixOSM0uLUkpLiHayMAITqL8lhAAAA&sa=X&ved=2ahUKEwjZ7dOu7YT0AhU1VTABHa_CAAsQmxMoAXoECFMQAw), USA |
| **Anti-rabbit IgG, HRP-linked Antibody** | Cell Signaling Technology | [Danvers, MA](https://www.google.com/search?rlz=1C1CHBF_enUS878US878&sxsrf=AOaemvIga4EeEji4x9E7mXFgVRfe0zLDKQ:1636240098401&q=Danvers,+Massachusetts&stick=H4sIAAAAAAAAAOPgE-LSz9U3MCu3KKlIUeIEsQ1zKsrztLSyk63084vSE_MyqxJLMvPzUDhWGamJKYWliUUlqUXFi1jFXBLzyoAsHQXfxOLixOSM0uLUkpLiHayMAITqL8lhAAAA&sa=X&ved=2ahUKEwjZ7dOu7YT0AhU1VTABHa_CAAsQmxMoAXoECFMQAw), USA |
| **Aldefluor™ Kit Stem Cell Technologies** | Stem Cell Technologies | Vancouver, Canada |
| **CellTiter-Glo 2.0 Assay** | Promega | Madison, WI, USA |
| **Caspase-Glo® 3/7 Assay System** | Promega | Madison, WI, USA |
| **Bortezomib** | Selleck Chemicals | Houston, TX, USA |
| **Ibrutinib** | Selleck Chemicals | Houston, TX, USA |

**Table-S2. Summary of Combination Index (CI) and Dose Reduction Index (DRI) values corresponding to the combination experiments shown in Figures 1B–E.** The Combination Index (CI) and Dose Reduction Index (DRI) values were calculated by Calcusyn Software (BioSoft) using Chou-Talalay's algorithm (CI<0.9 indicates drug synergy).

|  | **YM155+IBR** | | | | **S63845+IBR** | | | | **YM155+BTZ** | | | | **S63845+BTZ** | | | |
| --- | --- | --- | --- | --- | --- | --- | --- | --- | --- | --- | --- | --- | --- | --- | --- | --- |
|  | **MINOP** | **MINOVR** | **JEKO1** | **Z-138** | **MINOP** | **MINOVR** | **JEKO1** | **Z-138** | **MINOP** | **MINOVR** | **JEKO1** | **Z-138** | **MINOP** | **MINOVR** | **JEKO1** | **Z-138** |
| **CI_Min_** | 0.02 | 0.02 | 0.10 | 0.24 | 0.02 | 0.01 | 0.12 | 0.00 | 0.11 | 0.08 | 0.02 | 0.80 | 0.15 | 0.01 | 0.05 | 0.00 |
| **DRI_Max_** | 106.1 | 740.5 | 176.5 | 1560.7 | 1928.4 | 629.1 | 3086.9 | 2536.4 | 10.9 | 2041.7 | 1338.2 | 10.1 | 17.8 | 10200.0 | 274.5 | 1500.4 |
| **DRI_Median_** | 6.8 | 84.2 | 13.7 | 131.7 | 62.6 | 24.1 | 87.0 | 73.9 | 3.6 | 1028.8 | 167.3 | 1.2 | 4.9 | 2050.5 | 22.5 | 119.7 |
| **DRI_Mean_** | 22.9 | 157.8 | 35.4 | 343.2 | 271.9 | 106.1 | 466.0 | 375.9 | 4.1 | 1051.5 | 341.9 | 1.9 | 7.3 | 3257.6 | 51.3 | 325.6 |

**Table-S3. Top 50 differentially expressed genes (DEGs) for each single-agent secDrug treatment**

**S3A. YM155**

| **Gene name** | **P-value (YM155 vs CON)** | **Fold change (YM155 vs CON)** |
| --- | --- | --- |
| **INSM1** | 1.12E-04 | -5.858 |
| **SDC1** | 4.02E-02 | -5.829 |
| **CTXN1** | 1.16E-03 | -5.666 |
| **FAM72D** | 1.17E-02 | -4.922 |
| **SPINT2** | 6.28E-03 | -4.675 |
| **SYNPO** | 7.95E-03 | -4.525 |
| **CENPV** | 2.27E-02 | -4.525 |
| **GAMT** | 3.06E-03 | -4.505 |
| **PXMP2** | 1.32E-03 | -4.340 |
| **SIT1** | 5.55E-04 | -4.248 |
| **PIMREG** | 3.53E-04 | -4.247 |
| **AURKB** | 8.29E-04 | -4.167 |
| **NEIL3** | 1.20E-02 | -4.121 |
| **FAM72A** | 2.01E-02 | -4.024 |
| **ACSM3** | 3.53E-02 | -3.905 |
| **ATOH8** | 6.22E-03 | -3.813 |
| **ABCG1** | 3.47E-02 | -3.786 |
| **VASH2** | 1.83E-02 | -3.715 |
| **SPIB** | 2.74E-02 | -3.489 |
| **KCNA3** | 2.55E-02 | -3.417 |
| **SCAMP5** | 2.05E-02 | -3.397 |
| **SDHAF4** | 2.29E-02 | -3.395 |
| **TERT** | 2.95E-03 | -3.351 |
| **RASL10B** | 4.73E-02 | -3.205 |
| **CD72** | 1.43E-02 | -3.118 |
| **STXBP1** | 2.97E-02 | 2.640 |
| **PSAP** | 3.93E-02 | 2.697 |
| **BHLHE40** | 3.07E-06 | 2.863 |
| **HCN2** | 2.20E-02 | 2.908 |
| **RHOQP2** | 7.77E-03 | 3.149 |
| **SKIL** | 6.61E-03 | 3.245 |
| **DUSP10** | 2.09E-02 | 3.361 |
| **SDCBP** | 7.13E-04 | 3.396 |
| **NEU1** | 5.85E-04 | 3.437 |
| **SNX30** | 2.26E-04 | 3.793 |
| **GADD45G** | 1.54E-02 | 3.946 |
| **CAMKK1** | 2.51E-03 | 3.992 |
| **STX3** | 2.02E-05 | 4.067 |
| **LUCAT1** | 3.25E-03 | 4.742 |
| **TRPV3** | 3.69E-02 | 4.997 |
| **SQSTM1** | 3.47E-03 | 5.394 |
| **LINC03016** | 3.49E-03 | 5.593 |
| **EPHX1** | 5.63E-04 | 6.213 |
| **LMNA** | 8.56E-03 | 6.990 |
| **NQO1** | 7.38E-04 | 7.823 |
| **RGS1** | 2.67E-04 | 8.424 |
| **OSGIN1** | 5.57E-03 | 9.426 |
| **GPNMB** | 3.28E-04 | 10.734 |
| **UPP1** | 7.34E-04 | 10.983 |
| **SERPINE1** | 6.59E-03 | 12.723 |

**S3B. S63485.**

| **Gene name** | **P-value (S63845 vs CON)** | **Fold change (S63845 vs CON)** |
| --- | --- | --- |
| **ABCG1** | 6.65E-03 | -4.740 |
| **STAC3** | 4.83E-03 | -4.286 |
| **NYNRIN** | 2.17E-03 | -3.012 |
| **ATP7B** | 4.54E-02 | -2.911 |
| **IGHD** | 2.66E-02 | -2.799 |
| **ADAMTS6** | 3.89E-02 | -2.754 |
| **PVRIG** | 2.61E-02 | -2.639 |
| **PAOX** | 4.96E-02 | -2.613 |
| **LCK** | 3.49E-02 | -2.588 |
| **IL10** | 3.38E-02 | -2.586 |
| **PIF1** | 3.14E-02 | -2.343 |
| **KREMEN1** | 4.25E-02 | -2.274 |
| **REEP2** | 2.02E-02 | -2.263 |
| **CCDC28B** | 1.98E-02 | -2.231 |
| **ICA1** | 4.44E-02 | -2.208 |
| **SGCA** | 4.19E-02 | -2.158 |
| **KLHL13** | 7.83E-03 | -2.136 |
| **WNK2** | 4.97E-03 | -2.109 |
| **HMGA1P2** | 7.02E-03 | -2.083 |
| **DLGAP3** | 5.95E-03 | -2.074 |
| **TESMIN** | 2.18E-02 | -2.033 |
| **ZEB1-AS1** | 2.41E-02 | -2.032 |
| **OTX1** | 5.01E-03 | -2.028 |
| **SNHG1** | 2.92E-02 | -1.965 |
| **TMEFF1** | 4.48E-02 | -1.855 |
| **UBE2L5** | 2.63E-02 | 1.483 |
| **CYP51A1** | 3.17E-02 | 1.626 |
| **USP34-DT** | 2.56E-02 | 1.685 |
| **MTND5P11** | 4.64E-02 | 1.828 |
| **PNLDC1** | 2.48E-02 | 1.830 |
| **KIAA1614-AS1** | 1.78E-02 | 1.962 |
| **LINC02908** | 6.19E-03 | 2.112 |
| **CLEC2B** | 1.09E-02 | 2.175 |
| **QPRT** | 1.25E-02 | 2.338 |
| **EPHX1** | 3.15E-02 | 2.440 |
| **FTLP3** | 3.77E-02 | 2.522 |
| **MT-CYB** | 3.62E-02 | 2.570 |
| **SIGLEC15** | 4.08E-02 | 2.655 |
| **MAST4** | 2.96E-02 | 2.716 |
| **SAMD11** | 3.11E-02 | 2.788 |
| **LINC01484** | 4.62E-02 | 3.137 |
| **SQSTM1** | 3.33E-02 | 3.158 |
| **MT-ND2** | 3.45E-02 | 3.168 |
| **GADD45G** | 1.51E-02 | 3.350 |
| **PLCH2** | 3.80E-02 | 3.359 |
| **FBXO32** | 3.19E-02 | 4.577 |
| **NQO1** | 8.49E-04 | 7.236 |
| **HMOX1** | 1.33E-03 | 11.611 |
| **MT-RNR1** | 3.75E-02 | 16.390 |
| **SNORD3A** | 1.41E-02 | 246.573 |

Table-S4. Top DEGs for each treatment combination in the sensitive line MINO-P (A. YM155+IBR, B. S63845+IBR) and the resistant line MINO-VR: (C. BTZ+YM155, D. BTZ +S63845).

S4.A. IBR+YM155 Combination

| **Gene name** | **P-value (MINO-P,IBR_YM155 vs MINO-P,CON)** | **Fold change (MINO-P, IBR_YM155 vs MINO-P, CON)** |
| --- | --- | --- |
| **CD72** | 1.74E-02 | **-15.21** |
| **TERT** | 6.54E-05 | **-14.26** |
| **SPIB** | 3.98E-02 | **-12.35** |
| **ABCG1** | 1.82E-03 | **-11.91** |
| **SIT1** | 2.21E-03 | **-10.37** |
| **GAMT** | 1.20E-02 | **-8.79** |
| **NDC80** | 1.93E-02 | **-8.50** |
| **ACSM3** | 1.56E-02 | **-7.12** |
| **NEIL3** | 3.59E-02 | **-6.75** |
| **CTXN1** | 5.70E-03 | **-6.54** |
| **SCAMP5** | 3.29E-03 | **-6.45** |
| **SDHAF4** | 2.14E-03 | **-6.30** |
| **HPDL** | 2.89E-02 | **-6.14** |
| **SPINT2** | 2.55E-02 | **-5.89** |
| **DNPH1** | 2.61E-02 | **-5.45** |
| **RASL10B** | 4.28E-03 | **-5.31** |
| **AURKB** | 6.85E-03 | **-5.20** |
| **INSM1** | 4.68E-02 | **-5.03** |
| **HILPDA** | 3.23E-02 | **-4.81** |
| **CENPV** | 7.59E-03 | **-4.73** |
| **COMTD1** | 3.89E-02 | **-4.47** |
| **APRT** | 2.02E-02 | **-3.97** |
| **SDC1** | 4.89E-02 | **-3.57** |
| **BHLHE40** | 1.70E-02 | **2.44** |
| **NEU1** | 6.04E-03 | **3.91** |
| **SDCBP** | 2.34E-03 | **4.09** |
| **STXBP1** | 2.41E-02 | **4.59** |
| **SNX30** | 7.90E-03 | **5.39** |
| **STX3** | 7.88E-04 | **7.78** |
| **CAMKK1** | 1.84E-02 | **9.19** |
| **SQSTM1** | 1.33E-02 | **12.03** |
| **EPHX1** | 4.82E-03 | **12.69** |
| **GADD45G** | 3.05E-02 | **12.89** |
| **RGS1** | 4.50E-02 | **15.93** |
| **UPP1** | 3.85E-02 | **17.67** |
| **LUCAT1** | 5.88E-06 | **21.88** |
| **LINC03016** | 1.04E-03 | **23.44** |
| **NQO1** | 3.21E-03 | **25.33** |
| **SERPINE1** | 2.47E-02 | **30.47** |
| **OSGIN1** | 2.90E-03 | **50.24** |

S4.B. IBR+S63845 Combination

| **Gene name** | **P-value (MINO-P, IBR_S63845 vs MINO-P, CON)** | **Fold change (MINO-P, IBR_S63845 vs MINO-P, CON)** |
| --- | --- | --- |
| **ABCG1** | 3.33E-04 | **-20.794** |
| **LCK** | 9.13E-03 | **-13.473** |
| **LINC02642** | 7.07E-03 | **-12.108** |
| **ADAMTS6** | 2.81E-03 | **-9.645** |
| **NIPSNAP1** | 3.00E-06 | **-9.554** |
| **PIF1** | 2.71E-02 | **-9.314** |
| **CCDC28B** | 6.38E-03 | **-8.513** |
| **TMEFF1** | 2.23E-02 | **-7.645** |
| **PVRIG** | 1.94E-03 | **-7.229** |
| **KLHL13** | 2.26E-08 | **-6.866** |
| **PAOX** | 2.12E-02 | **-6.235** |
| **LYAR** | 1.94E-03 | **-5.938** |
| **ICA1** | 8.68E-03 | **-5.306** |
| **DLGAP3** | 1.45E-04 | **-5.022** |
| **ATP7B** | 2.30E-02 | **-5.006** |
| **APOM** | 2.34E-02 | **-4.471** |
| **PACC1** | 4.33E-03 | **-4.223** |
| **WNK2** | 1.98E-03 | **-4.179** |
| **HMGA1P2** | 4.70E-02 | **-3.758** |
| **REEP2** | 4.53E-02 | **-3.614** |
| **MIPEP** | 8.07E-03 | **-3.564** |
| **ESD** | 2.83E-02 | **-3.477** |
| **CD4** | 3.28E-03 | **-3.434** |
| **ECE1-AS1** | 2.47E-06 | **-3.247** |
| **PEX7** | 4.98E-02 | **-2.829** |
| **EIF5AP4** | 3.26E-02 | **-2.714** |
| **RASA4DP** | 1.59E-03 | **-2.709** |
| **RDM1** | 1.09E-02 | **-2.444** |
| **OTX1** | 1.95E-02 | **-2.435** |
| **SLC2A4** | 3.22E-04 | **-2.435** |
| **ATP5F1C** | 1.69E-02 | **-2.331** |
| **PKD1L2** | 1.33E-02 | **-2.311** |
| **RHPN1-AS1** | 1.90E-03 | **-2.297** |
| **RDM1P5** | 9.94E-06 | **-2.061** |
| **PPP1R1B** | 2.99E-03 | **-2.061** |
| **VCAN** | 5.09E-04 | **-1.874** |
| **SNX5P1** | 8.51E-03 | **-1.812** |
| **LINC03062** | 2.41E-04 | **-1.587** |
| **ASIC1** | 3.28E-03 | **-1.437** |
| **RPL29P20** | 6.95E-05 | **-1.437** |
| **CD3E** | 3.60E-02 | **-1.437** |
| **MCIDAS** | 6.55E-04 | **-1.437** |
| **RAC1P4** | 3.44E-03 | **-1.374** |
| **CDKL4** | 1.25E-02 | **-1.187** |
| **MIR130AHG** | 5.23E-03 | **-1.187** |
| **GUCY2F** | 1.91E-02 | **-1.187** |
| **PRRT1B** | 5.52E-03 | **1.542** |
| **MSTO2P** | 9.32E-05 | **1.734** |
| **QPRT** | 1.90E-02 | **3.773** |
| **SIGLEC15** | 9.54E-03 | **5.154** |
| **CLEC2B** | 9.80E-05 | **7.138** |
| **FBXO32** | 8.14E-04 | **8.567** |
| **MT-ND2** | 4.70E-02 | **10.638** |
| **EPHX1** | 5.49E-03 | **12.078** |
| **GADD45G** | 2.98E-02 | **13.073** |
| **MT-CYB** | 2.05E-02 | **13.592** |
| **SQSTM1** | 8.71E-03 | **14.503** |
| **LINC01484** | 2.36E-03 | **15.003** |
| **MT-RNR1** | 1.75E-02 | **27.136** |
| **NQO1** | 1.88E-03 | **32.298** |
| **HMOX1** | 9.12E-03 | **48.832** |
| **SNORD3A** | 1.32E-02 | **156.844** |

S4.C. BTZ+YM155 Combination

| **Gene name** | **P-value (MINO-VR, BTZ_YM155 vs MINO-VR, CON)** | **Fold change (MINO-VR, BTZ_YM155 vs MINO-VR, CON)** |
| --- | --- | --- |
| **TERT** | 1.93E-05 | **-19.82** |
| **SPIB** | 1.99E-02 | **-18.38** |
| **FAM72A** | 4.82E-02 | **-15.98** |
| **HPDL** | 2.69E-03 | **-14.90** |
| **KCNA3** | 2.49E-02 | **-14.69** |
| **FAM72D** | 1.96E-02 | **-14.59** |
| **GAMT** | 4.64E-03 | **-12.56** |
| **INSM1** | 4.56E-03 | **-12.07** |
| **CD72** | 2.84E-02 | **-11.82** |
| **ACSM3** | 4.99E-03 | **-10.69** |
| **TMPO-AS1** | 1.83E-02 | **-9.08** |
| **PIMREG** | 1.74E-02 | **-8.55** |
| **NEIL3** | 2.66E-02 | **-7.68** |
| **NDC80** | 2.73E-02 | **-7.36** |
| **PXMP2** | 2.37E-02 | **-7.30** |
| **SDC1** | 5.95E-03 | **-6.72** |
| **CENPV** | 2.18E-03 | **-6.44** |
| **AURKB** | 3.77E-03 | **-6.07** |
| **SCAMP5** | 9.16E-03 | **-4.93** |
| **SDHAF4** | 5.91E-03 | **-4.91** |
| **ABCG1** | 3.72E-02 | **-4.45** |
| **CTXN1** | 2.46E-02 | **-4.28** |
| **HILPDA** | 4.70E-02 | **-4.22** |
| **CADM4** | 2.45E-02 | **-4.03** |
| **SDCBP** | 1.88E-02 | **2.75** |
| **BHLHE40** | 6.55E-03 | **2.85** |
| **NEU1** | 9.57E-03 | **3.55** |
| **STX3** | 5.29E-03 | **4.91** |
| **PSAP** | 4.01E-02 | **6.51** |
| **LUCAT1** | 4.60E-04 | **7.45** |
| **NQO1** | 2.36E-02 | **10.16** |
| **LINC03016** | 9.13E-03 | **10.21** |
| **SQSTM1** | 5.59E-03 | **17.61** |
| **UPP1** | 3.66E-02 | **18.27** |
| **GADD45G** | 4.01E-03 | **38.13** |
| **OSGIN1** | 3.08E-03 | **48.69** |

S4.D. BTZ+S63845 Combination

| **Gene name** | **P-value (MINO-VR, BTZ_S63845 vs MINO-VR, CON)** | **Fold change (MINO-VR, BTZ_S63845 vs MINO-VR, CON)** |
| --- | --- | --- |
| **IL10** | 3.38E-03 | **-29.246** |
| **CCDC28B** | 1.55E-03 | **-13.639** |
| **IGHD** | 8.10E-03 | **-12.100** |
| **LINC02642** | 1.45E-02 | **-9.111** |
| **ATP7B** | 7.11E-03 | **-7.269** |
| **ICA1** | 2.77E-03 | **-7.239** |
| **LYAR** | 1.49E-03 | **-6.322** |
| **ABCG1** | 2.49E-02 | **-5.093** |
| **ADAMTS6** | 4.66E-02 | **-3.949** |
| **PVRIG** | 3.09E-02 | **-3.500** |
| **CD4** | 3.30E-03 | **-3.430** |
| **NIPSNAP1** | 2.06E-03 | **-3.167** |
| **RSPH1** | 3.07E-03 | **-3.136** |
| **WNK2** | 9.17E-03 | **-3.132** |
| **RSPH14** | 4.87E-03 | **-3.061** |
| **PACC1** | 2.91E-02 | **-2.810** |
| **RASA4DP** | 1.30E-03 | **-2.780** |
| **SLC2A4** | 1.06E-03 | **-2.171** |
| **CFAP58** | 1.68E-02 | **-2.156** |
| **GUCY2F** | 2.22E-08 | **-2.031** |
| **KANK3** | 9.98E-03 | **-1.945** |
| **MST1P2** | 7.31E-04 | **-1.907** |
| **LINC02321** | 3.18E-02 | **-1.890** |
| **RDM1P5** | 5.94E-05 | **-1.843** |
| **MANSC1** | 6.14E-03 | **-1.776** |
| **TLK1P1** | 1.37E-02 | **-1.749** |
| **HIVEP2-DT** | 7.58E-04 | **-1.589** |
| **PFKFB1** | 3.76E-02 | **-1.576** |
| **KLHL13** | 2.42E-02 | **-1.562** |
| **VCAN** | 2.55E-02 | **-1.422** |
| **SINHCAFP1** | 1.36E-02 | **-1.375** |
| **MSTO2P** | 2.43E-02 | **-1.296** |
| **LINC03062** | 2.09E-02 | **-1.281** |
| **RPL29P20** | 2.04E-02 | **-1.187** |
| **CLEC2B** | 4.34E-03 | **3.490** |
| **QPRT** | 8.74E-04 | **8.161** |
| **NQO1** | 2.60E-02 | **9.717** |
| **FBXO32** | 9.89E-05 | **14.973** |
| **SQSTM1** | 3.92E-03 | **20.551** |
| **GADD45G** | 4.46E-03 | **36.037** |
| **HMOX1** | 4.32E-03 | **78.835** |
